# Observations on the Crural Hernia

**Published:** 1804-03-01

**Authors:** 


### OBSERVATIONS ON THE CRURAL HERNIA, BY DR. HULL.

( Continued from pp. 119—129. )

HAVING pointed out the different methods of dilating and dividing the crural ring, that have been proposed by the principal chirurgical writers of Britain and the Continent of Europe, I shall now proceed to give some directions for performing the whole of the operation for the incarcerated crural hernia, under the various circumstances in which this may be necessary.

I. *When*

---

I. *Where the hernia was perfectly reducible, previously to the incarceration, is of small size, and from the shortness of the period the incarceration has existed and the absence of alarming symptoms, there is great reason to believe the displaced bowel or bowels neither gangrenous nor on the point of becoming so.*

A. The taxis and other remedies having been diligently employed without success, the patient is to be placed upon a table of convenient height, or upon a bed; the external incision should be begun an inch or an inch and a half above the hernia, and continued to the same distance below it. This incision should be made in such a direction, as to pass over the middle of the tumour, and to intersect the crural arch at or nearly at right angles, and it should be continued through the skin and cellular membrane, avoiding, as much as possible, the lymphatic glands with which this is often beset.

The lower part of the tendon of the m. obliquus externus and the upper part of the fascia of the thigh being exposed, an incision is to be made with caution through the fascia, the whole length of the tumour.

An attempt may now be made to reduce the displaced parts, and if the reduction cannot be effected by the ap plication of moderate pressure, we should proceed to cut the crural arch.

With this view, a small aperture is to be made through the tendon of the m. obliquus externus, about half an inch above the crural arch, at which the point of a grooved director is to be introduced, and being kept in close contact with the posterior surface of the tendon, passed down to the arch; this portion of tendon is then to be divided upon it by a probe-pointed bistourie with a narrow blade. An assistant is now to draw the spermatic cord upwards with a blunt hook, or bent probe, and the crural arch is to be cautiously divided from before backwards, till it be entirely cut through, or till the crural ring appear to be sufficiently enlarged; and in doing this, the edge of the knife should be turned towards that portion of the linea alba which lies betwixt the umbilicus and symphysis pubis, that the arch may be cut obliquely inwards, near to its insertion in the os pubis.

We have here been supposing that the patient is a male. In operating upon a female, we may either divide the tendon, as directed above, or, omitting this, may immediately divide the crural arch; as the round ligament of the womb womb may not be wounded, or, if wounded, no bad consequences may ensue. At least, I have cut through the crural arch in this way, without perceiving any hæmorrhage or other accident from it in the female.

By moderate pressure upon the hernial sac we should now endeavour to return its contents into the abdomen.

If we succeed in this, the wound may be considered as a simple incised wound; its lips are to be brought into close contact, and secured by the interrupted suture and a sufficient number of straps of adhesive plaster. Over these a pledgit of lint spread with cerate and tow is to be applied and retained by a proper bandage. The patient is to be placed in bed with the hips raised as high, or higher than the head, a strict antiphlogistic regimen is to be observed, and saline cathartics are to be employed, &c. &c.

B. If the reduction of the bowel be found impracticable after the division of the crural arch, and the hernia be of long standing, there is reason to suspect a stricture formed by the neck of the sac. To remove this, a small opening is to be made through the sac, either by cutting carefully down through it, and frequently examining with a probe or director, to ascertain when the sac is cut thro'; or by raising a small portion of the sac with a pair of dissecting forceps, and cautiously cutting this horizontally, the side of the knife being turned to the sac. A slender director being introduced at this opening, the neck of the sac is to be divided upon it. Should it be difficult to execute the division of the sac in this way with safety, a probe bent into a semicircle at the point is to be introduced at the opening, and the point being carried beyond the stricture is to be made to press upon the peritonæum, which is to be cut upon the end of the probe. The probe being then pushed through the upper opening, the neck of the sac may be divided upon it without any danger of wounding the contents. If the sac be thick and opake, or dark-coloured, and there be a suspicion that the bowels may adhere to it, Dr. Monro* with great propriety, advises us to make the first opening in the peritonæum above the stricture, then to introduce a probe bent near its point into a semicircle directly downwards through the stricture into the sac, and upon the point to make another small hole; after which we may either cut the intervening portion of the

* Description of all the Bursæ Mucosæ, &c. p. 52.

the sac upon the probe, or may introduce a furrowed directory, and divide the neck of the sac upon it.

The neck of the sac being divided, the intestine or omentum may be returned, provided this has been the seat of the stricture.

C. If the reduction of the bowel should still be prevented, it is to be suspected, that the sac and its contents adhere, and, as the adhesion must be recent and of the spongy kind, it will be proper to lay the sac completely open, to separate the bowel from it by a finger, or probe, and then reduce it.

The wound being now properly cleaned, the edges of the sac are to be laid together and the divided integuments are to be secured in contact by the interrupted suture, &c. taking care not to pass the ligatures through the edges of the sac.

As the method of operating in crural hernia, without opening the sac, is not so generally adopted as it seems to deserve, I shall in this place make a few observations on the advantages arising from this practice, where it is properly applicable, and succeeds as in the case stated above.

Three sources of danger, arising from the operation as performed in the usual way, are avoided. 1st. The peritonæum is not wounded, consequently the danger arising from this cause of inflammation is avoided. 2. Neither the prolapsed parts, nor the internal surface of the peritonæum, are exposed to the air. 3. The danger of wounding the bowels, which is incurred by opening the sac, is completely avoided: and the greatest nicety of the operation, in many cases, consists in opening the sac.

Where the operation is performed without opening the sac, provided the arteria obturatoria is in its natural situation, the danger is confined to the wound of the common integuments and the crural arch, and is scarcely greater in my opinion than that arising from the degree of force commonly employed in the taxis.

This practice appears more particularly applicable to the crural than the inguinal hernia, because the former is almost always of small size, and therefore strictures are less liable to be formed amongst the contents of the sac. Great danger has been apprehended by some writers from returning the serous fluid, effused within the sac, into the abdomen. From this circumstance I should expect no danger, and those who are of a different opinion, must perceive that the danger to be apprehended from this source is very trifling, as the crural hernia is commonly not not larger than a walnut and often as small as a hazel nut.

According to Sabatier, both Franco and Paré practised the method of operating in hernia without opening the sac. Mr. Petit practised it before the year 1720, and in his posthumous work has replied to the objections that had been urged against it. The following quotation will show in what cases he deemed this method of operating inadmissible, and that he was well acquainted with the advantages resulting from it in those cases, wherein he had recommended it. "Mon sentiment est donc, qu' excepté les hernies gangréneuses, celles qui sont maronnées & quelques-unes de celles, dans lesquelles l'intestin contient des corps étrangers, toutes les autres peuvent etre traitées ainsi; il y en a même, qu'on ne doit point traiter autrement."

"Qu'on se demande à soi-même quelle utilité il y a d'ouvrir le sac? Je n'en connois aucune, si ce n'est de découvrir l'intestin & l'epiploon, pour remédier à leurs altérations, supposé qu'il y en ait, de le détacher de lui-même & de l'épiploon dans les hernies maronnées, & de pouvoir toucher immédiatement l'intestin, pour faire rentrer les matières fécales endurcies & même les corps étrangers, s'il y en étoit. Or ces trois cas sont ceux, que j' excepte: dans tous les autres, qui sont en bien plus grande nombre, pourquoi ouvrir le sac? Rien ne nous indique de le faire; il est même très avantageux d'eviter cette opération, *parceque l'on n'expose point les parties à l'air*; *on ne court point risque de les blesser en ouvrant le sac*, & de plus je ferai voir ailleurs, que pour les suites, il est bien plus avantageux, que le sac n'ait souffert aucune solution de continuité. De tout cela, je tire cette consequence, qu'il vaut mieux débrider l'anneau par le dehors, que par le dedans du sac." *Traité des Maladies Chirurgicales*, t. ii. p. 373.

This mode of performing the operation is particularly recommended by Dr. Monro, in his "Description of all the Bursæ Mucosæ," p. 43, &c. who has adopted the opinion and reasoning of Mr. Petit, and confirmed the propriety of these by the relation of different cases. In a Note to p. 57, the Doctor has the following observation. "We will readily perceive, that Mr. Petit did not perceive the chief advantages of the operation he proposed; I mean, that the danger in the common method of operating arises chiefly from the exposure of the bowels to the air; and as this material fact has been little better understood by various authors who have lately written on the subject, subject, that method has been rejected by them on as slight foundation as that on which it was proposed by Mr. Petit." But it appears from the above quotation, that Mr. Petit has particularly noticed the advantage of not exposing the parts to the air.

The incarcerated crural hernia is a frequent and dangerous complaint. The danger of the disease, as well as the difficulty of the operation, which is required for its removal, are confessedly increasing every hour. It therefore is the duty of the surgeon, when called in at the commencement of the complaint, to make an immediate and fair trial of the remedies that are most powerful in taking off the strangulation; and where these do not prove successful, to propose the operation without delay. When an operation can be considered by a surgeon, and recommended to a patient, as not attended with much danger, pain, or difficulty, and as affording a very good chance of recovery from a disease extremely dangerous in itself, it will be more likely to be proposed by the one and submitted to by the other at a sufficiently early period to be attended with success. In this point of view the operation in question may, I conceive, very properly be regarded, and may, if generally adopted, be the means of preserving many valuable lives that would otherwise be lost.

II. *Where the hernia was perfectly reducible before the incarceration, but from the long continuance of this and especially from the violence of the symptoms, there is great reason to fear that the prolapsed parts are either gangrenous or tending strongly to gangrene.*

Under these circumstances it would be evidently improper to attempt the reduction of the bowel or bowels, without opening the sac, since these, if mortified, must separate, and may thereby occasion so much mischief in the cavity of the abdomen as to prove fatal. It is therefore necessary to open the sac, and examine the state of its contents, which should be done before the crural ring is divided.

With this view, a small opening is to be made into the sac, in one of the ways mentioned above; and it is in general better to make this near the bottom of the sac. The opening is to be dilated by a probe-pointed bistouri either introduced upon a grooved director or guided by the fingers up to the anterior margin of the crural arch.

A. If the prolapsed viscus, or viscera, though inflamed or discoloured should neither adhere to the sac, nor be affected fected with gangrene, there can be no doubt with respect to the propriety of returning it. We have been advised by some writers to pull down an additional portion of bowel into the sac, before we cut the crural arch, as the division of the arch may thus be rendered unnecessary. This attempt, however, should be made with great care, since any material force applied to the bowels, would be productive of more danger than the division of the arch, and would often fail of success; and I consider it as a better general rule, to proceed to the division of the crural arch immediately after we have determined upon reducing the contents of the sac.

B. Should the bowel or bowels adhere to the sac, and not be gangrenous, in all probability the adhesion will be of the spongy kind, formed by a layer of coagulable lymph, either wholly inorganic or but imperfectly organized, and may be detached without the use of the knife, by the means recommended above. In this case, therefore, we may separate the prolapsed parts from the sac, and then reduce them.

C. If the hernia be an Epiplocele, and the displaced portion of omentum be in a gangrenous state, this is to be carefully unfolded, and removed by a pair of scissars. Should any vessel or vessels bleed, these are to be separately secured by ligatures, which should be left of a sufficient length to hang out of the wound, that they may be taken away after they have separated. No ligature, as was formerly practised, should be tied tight round the whole of the protruded omentum.—If the omentum be in a dubious state, or if the limits betwixt the gangrenous and living parts be not well marked, it may be advisable to leave it in the sac.

D. If the hernia be of the intestinal kind, and the prolapsed portion should be merely an appendix, as the appendicula vermiformis, or a preternatural elongation, forming a cul de sac, and gangrenous, this is to be removed by a pair of scissars. The upper portion, if its diameter be considerable, and especially if fæces be discharged from it, should be stitched, before it is returned, in order to prevent the inconvenience and danger arising from the discharge of fæces from the wound, or into the abdomen. The ligatures here also should be left of sufficient length to be withdrawn after their separation.

E. When the hernia is an Enterocele, and the prolapsed portion, constituting a part of the regular alimentary canal,

 is is in a gangrenous state, we must proceed differently, according to the extent of the gangrene.

If the gangrene occupy the cæcum, or only a portion of the parietes of the intestine, the posterior or mesenteric part being situated above the stricture, it will be necessary, after opening the sac and dividing the crural arch, to apply the proper dressings; in doing which we are to take care that the wounds of the sac and integuments do not heal by the first intention; that the fæces, discharged through the opening in the intestine, are frequently removed; and that the strength of the patient is supported by nutritious clysters, if a great part of the aliment, taken into the stomach, pass through the wound. In several cases of this kind the aperture in the intestine has gradually diminished and at length entirely healed, and the discharge from the wound has gradually diminished till at length the whole of the fæces have passed by the anus. In other cases, a portion of the fæces continue to be discharged at the groin, the sore remaining fistulous: or the whole of the fæces pass this way, and then the orifice is to be regarded as an artificial anus, and is to be prevented from closing by the introduction of a tent. In instances of this kind it is desirable to know, whether any part of the food taken by the mouth passes through the whole tract of the intestines, and for this purpose currants will be found a very convenient test. These being of small size, and indigestible, will be passed per annus, if not wholly discharged from the opening occasioned by the hernia. By some writers we are advised not to cut the crural arch in these cases, for fear of destroying the adhesion by which the intestine is attached to the parts about the ring: But the mere division of the arch would not, I apprehend, in any instance separate the intestine when it does adhere; and if it should not adhere, the division of the arch would be necessary to enable us to pass a needle and ligature through the mesentery, with the view of fixing the intestine in the wound.

If the whole of the parietes of the prolapsed intestine be affected with gangrene, we should divide the crural arch, and then endeavor to draw down a portion of the sound intestine, connected with each end of the gangrenous part, into the sac. If this should be impracticable, in consequence of adhesions at or above the neck of the sac, the operator can do no more than apply the dressings in such a manner as to admit of the fæces being discharged by the wound. It is scarcely to be expected, that under these circumstances, circumstances, the divided extremities of the intestine can unite in such a manner as to restore the continuity of the alimentary canal. In general, if the life of the patient be preserved, it must be by submitting to the inconvenience of an artificial anus.

When a portion of sound intestine, connected with each extremity of the part that is become gangrenous, is pushed down, or can be pulled down, after the crural arch has been cut through, a choice of two modes of proceeding is left to the operator. He may either content himself with establishing an artificial anus, or he may endeavour to unite the sound ends of the intestine, after removing the mortified portion.

To form an artificial anus, it is necessary to secure both the divided extremities in the wound by passing a ligature through the mesentery, and fastening this to the thigh by a piece of adhesive plaster; because we are not able to determine with certainty which end is connected with the upper part of the alimentary canal. After this has been ascertained, by administering oil or currants, it will not be requisite to detain the extremity of the inferior portion of the intestinal canal in the wound any longer. It may therefore be returned into the abdomen, after clearing it by the injection of some warm water, or after stitching up the aperture, if this should appear necessary from any discharge that may take place from it. The discharge of the fæces from the remaining end of the intestine, is then to be provided for by proper dressings, &c.

An artificial anus, with every advantage to be derived from an appropriate apparatus and bandage, proves a constant source of distress from its dirtiness; it is also liable to become extremely painful and even dangerous, in consequence of an inversion and protrusion of the intestine, resembling a common prolapsus ani; and in some cases an anus of this kind proves a cause of dangerous and fatal debility, the food passing off by it, before the chyle has been absorbed in sufficient quantity to answer the purposes of nutrition. This last inconvenience is unavoidable, where the portion of intestinal canal betwixt the stomach and the aperture, constituting the anus, is of short extent. To avoid this source of inconvenience and danger, it has been proposed to stitch the sound extremities of intestine together, after the removal of the gangrenous portion, and in a few instances this has been put in practice with success. With this view the extremity of the superior portion of intestine has been introduced into the extremity of the inferior portion, and these have been sewed together without introducing any extraneous substance into the intestine. In other cases, a piece of the trachea of some animal, or a hollow cylinder formed of a piece of card or thin pasteboard, or a piece of a tallow candle of a proper thickness, have been introduced into both extremities of the intestine, before the needle and ligature have been employed to sew them together. An obvious advantage is to be derived from interposing some substance of this kind, before we attempt to stitch the intestine, viz. that we run no risk of sewing the opposite sides of the intestine together, when we only wish to unite the two ends.

F. Another case has occurred, wherein the prolapsed portion of intestine, though not gangrenous, must necessarily be cut off, and treated in one of the ways just described, in order to give the patient a chance for life: namely, where two points of intestine have been so long and so strongly compressed, that an adhesion of different parts of the internal coat has taken place and obliterated the canal. The intestine contained in a herniary sac has frequently been observed to be much contracted in the points where it has sustained the pressure of the ring, but I should suppose it has rarely been rendered impervious from this cause, though it must be regarded as a possible case, and consequently as deserving the attention of the operator. The pervious, or impervious state of the intestine in these points, may upon some occasions be ascertained by pressing, or attempting to press, the contents of the intermediate portion of the intestine through them, or by pulling the parietes gently asunder. Where the canal of the intestine is pervious, we need not hesitate about the reduction, as we may expect the contracted points will gradually recover their natural diameters.

G. If both a portion of omentum and intestine should be found affected by gangrene on opening the herniary sac, the operation is to be conducted according to the directions given above for the management of the cases, wherein these states of the omentum and intestine occur singly.

III. *Where the contents of the sac were irreducible for a considerable time previously to the incarceration, and there is every reason to believe them to be neither gangrenous nor on the point of becoming so.*

A hernia may be rendered irreducible independently of incarceration, from the bulk or change of form of the displaced displaced parts, or from the adhesion of these to each other and to the sac.

It rarely happens, I believe, that a crural hernia becomes irreducible merely from the bulk or change of form of the contents of the sac. Where a crural hernia is irreducible, before incarceration takes place, I should be induced to consider this as arising from adhesions, rather than from the bulk, or change of form of the bowels. Adhesions may affect the whole or only a portion of the contents of the sac, and hence the hernia may be wholly irreducible or partially reducible; and adhesions, when partial, may affect the neck of the sac only, or some part at a distance from the neck. Adhesions also differ in their nature, as well as in their extent and situation. Three kinds of adhesion have been described, viz.

1. The *spongy*, in which the parts are slightly glued together by a layer of coagulable lymph, and can easily be separated by the finger, or other blunt body pushed betwixt the adhering parts.

2dly. The *fibrous*, in which slender cords or filaments, of a softer or harder consistence, arise from one part of the contents or sac, and are attached to another.

3dly. The *fleshy* or *tendinous*, in which the parts are united together in such a manner, that they form an almost homogeneous mass, the constituent parts being scarcely, if at all, distinguishable from each other. The *spongy* adhesions are to be expected in herniæ, where the parts have only begun to adhere, after the incarceration has taken place. The *fibrous* and *fleshy* are to be expected, where the irreducible state has preceded the strangulation, and especially the latter, which is the worst kind of adhesion.

From a perusal of the writings of some celebrated surgeons, it would appear to be no difficult matter to separate any adhesions that may be met with betwixt a herniary sac and its contents. That the spongy kind of adhesions may be easily broke thro', I know from experience; and I also know from experience, as well as reading, how extremely embarrassing the fleshy kind of adhesions prove, and that it is impossible in some cases to separate these by dissection, without wounding either the prolapsed bowels or contiguous blood vessels.

A case of incarcerated Bubonocele, in which the hernia was of large size, had existed more than *sixty* years, and during a considerable part of the time had been in an irreducible state, occurred to Dr. Lancaster and myself. After trying the usual remedies, the operation was proposed and submitted to. On cutting the sac, which was very firm, thick and opake, we found the contents every where adhering to it, and the whole compacted into a mass, with scarcely any distinction of parts. With great difficulty we dissected the contents from the sac, and by carrying an incision thro' the inguinal ring as high as where the bowels passed out of the abdomen, we succeeded in returning them into the abdomen. No vessel was wounded in the operation, which required tying. Yet there is reason to believe, that the spermatic artery was divided, as the testicle and spermatic cord sloughed, and came away in a few days after the operation. It is remarkable, that, though we could not avoid wounding the omentum and intestine in this operation, the old man recovered without any dangerous symptom, and lived several years, following the occupation of a carpenter. This, I believe, was a case of hernia congenita, as we did not meet with a tunica vaginalis testis distinct from the sac, and the contents of the sac seemed to adhere immediately to the testicle.

Since I met with the above case, I have been of opinion, that the best general rule for performing the operation for an incarcerated crural hernia, that was previously irreducible, will be to divide the crural arch, and not meddle with the sac; unless, from its contracted and indurated state, the neck of the sac should prove or be suspected to be the seat of the stricture; we are in this case to cut through the neck of the sac in the manner laid down above, and may then desist from dividing the sac, if there should appear, from the introduction of a probe, to be any considerable adhesion betwixt the sac and its contents; or we may proceed to divide the sac through its whole length, if there be a prospect of reducing its contents without any material risk of injuring either these or the femoral vessels. As the patient may be deprived of the benefit of wearing a truss after the operation, if the prolapsed parts be not replaced, it is particularly desirable that they should be reduced, if this can be accomplished without increasing the danger. The operator should however recollect, that, from the depth which the crural hernia is situated, and its contiguity to the large femoral vessels, the scalpel cannot be used without great danger, to detach any adhesions betwixt the back-part of the herniary sac and its contents.

It may not be improper to mention here, that when an irreducible hernia passes into an incarcerated one, the incarceration may affect only the old prolapsed parts, or anadadditional portion of bowel, which does not adhere either to the sac or its contents, may be forced down into the sac, and become so far compressed as to occasion the symptoms of incarceration. In the latter instance the taxis may be resorted to with some prospect of success; and as we know no method of distinguishing these two cases, it would appear improper to omit it in any instance.

Whether the sac be opened or not, and whether the bowels be returned, or left in their protruded state, it will be proper to endeavour to heal the wound by the first intention, and therefore to bring the divided integuments into contact, and retain them by sutures and strips of adhesive plaster.

IV. *Where the contents of the sac were irreducible before they became incarcerated, and there is reason to believe these are already gangrenous.*

In this case it is not to be expected that an operation can prove of any use unless the sac be opened; therefore, after dividing the crural arch, a small aperture is to be made into the sac, which is afterwards to be continued through its whole length. If the sac and its gangrenous contents be found adhering strongly together, it will be better to leave these to separate, to keep the lips of the wound asunder, and apply the necessary dressings. If the prolapsed parts adhere but slightly to the sac, these may be cut off as near as possible to the sound.

Under these circumstances, if the prolapsed part be merely omentum or an intestinal appendix, the patient may recover perfectly. If the dead prolapsed part be only a portion of the anterior parietes of the intestine, or part of the cæcum, the recovery may also be complete. But, if a larger portion of intestine, and especially if its whole circumference should be grangrenous, we must expect that the patient, if he should recover, will have a fistula or artificial anus in the situation of the hernia.

V. *Where the contents of the herniary sac have been injured by external violence and incarceration, or symptoms resembling those of incarcerated hernia have taken place in consequence of the violence.*

A. If a person with a reducible crural hernia should receive a severe blow upon it, and should immediately have pain, swelling and tension in the part, together with violent sickness and vomiting, the displaced bowels not admitting of reduction by the taxis and remedies usually employed, the operation for the incarcerated crural hernia will will be necessary. In this case I should recommend opening the sac, although no symptoms of gangrene were present; because the contents may be so far injured as to render it improper to return them into the abdomen. But if the external injury, though it may have proved the exciting cause of the incarceration, have not been sufficient to immediately affect the texture of the bowels, the opening of the sac may not be necessary.

B. If a patient with an irreducible crural hernia should be exposed to a similar accident, and the same symptoms should immediately arise in consequence of it, and continue notwithstanding the use of the proper internal and external remedies, it would be equally necessary to operate in this case; but I think it would be better to divide the crural arch and leave the sac unopened, if there be reason to believe the contents were not already in a gangrenous state, or merely to divide the neck of the sac when that is suspected to be the seat of the stricture. But if there be reason to suspect the contents of the sac are mortified, this should be opened, since the operation without opening the sac would be of no avail in this case.

Hitherto I have not been consulted in any case of crural hernia, wherein the prolapsed parts had been injured by a severe blow: but I have lately had the care of a patient with a bubonocele, who had suffered from an accident of this kind. These cases prove particularly perplexing from our inability to determine how far the symptoms are occasioned by the injury immediately done to the parts by the contusion, or how far they are the consequence of a stricture induced on the parts, owing to their tumefaction.

At some future period I may publish this case, with some further remarks on Hernia, which want of leisure will not allow me to bring forward at present.

